# Electrostatic Interaction Between NS1 and Negatively Charged Lipids Contributes to Flavivirus Replication Organelles Formation

**DOI:** 10.3389/fmicb.2021.641059

**Published:** 2021-05-06

**Authors:** Yali Ci, Yang Yang, Caimin Xu, Cheng-Feng Qin, Lei Shi

**Affiliations:** ^1^State Key Laboratory of Medical Molecular Biology, Institute of Basic Medical Sciences, Chinese Academy of Medical Sciences and School of Basic Medicine, Peking Union Medical College, Beijing, China; ^2^Department of Biochemistry and Molecular Biology, Institute of Basic Medical Sciences, Chinese Academy of Medical Sciences and School of Basic Medicine, Peking Union Medical College, Beijing, China; ^3^State Key Laboratory of Pathogen and Biosecurity, Beijing Institute of Microbiology and Epidemiology, Beijing, China

**Keywords:** flavivirus, ER remodeling, replication organelles, PIP, non-structural protein 1

## Abstract

Flavivirus replication occurs in membranous replication compartments, also known as replication organelles (ROs) derived from the host ER membrane. Our previous study showed that the non-structural (NS) protein 1 (NS1) is the essential factor for RO creation by hydrophobic insertion into the ER membrane. Here, we found that the association of NS1 with the membrane can be facilitated by the electrostatic interaction between NS1 and negatively charged lipids. NS1 binds to a series of negatively charged lipids, including PI4P, and a positively charged residue, R31, located on the membrane-binding face of NS1, plays important roles in this interaction. The NS1 R31E mutation significantly impairs NS1 association with negatively charged membrane and its ER remodeling ability in the cells. To interfere with the electrostatic interaction between NS1 and negatively charged lipids, intracellular phosphatidylinositol phosphates (PIPs) level was downregulated by the overexpression of Sac1 or treatment with PI3K and PI4K inhibitors to attenuate flavivirus replication. Our findings emphasize the importance of electrostatic interaction between NS1 and negatively charged lipids in flavivirus RO formation.

## Importance

Flaviviruses have posed a great threat to human beings. However, the lack of effective antiviral drugs and vaccines for multiple flaviviruses argues the necessity of further exploring the flavivirus replication mechanism. The present study focuses on the biogenesis of flavivirus replication organelles, which is the essential structural basis for viral replication. We found that ZIKV NS1, the crucial factor to induce replication organelle, preferentially binds to negatively charged lipids, especially PIPs. The electrostatic interaction between NS1 and negatively charged lipids facilitates NS1 induced ER remodeling, which is essential to the formation of replication organelles. Downregulation of PIPs attenuated ZIKV replication, suggesting PIPs could be a promising antiviral target. Similarly, DENV and WNV NS1 proteins are inclined to interact with negatively charged lipids, implying a common rule among flaviviruses. This study reveals the important role of negatively charged lipids in flavivirus replication and deepens our understanding of the biogenesis of flavivirus ROs.

## Introduction

Positive strand RNA viruses, including flavivirus, coronavirus, and many others, are tightly associated with host cells’ membrane systems throughout their whole lifecycles, ranging from virus entry, fusion to replication, virions packaging, and secretion. These viruses replicate in the host membranous structure, which provides a unique environment and ensures that viral replication proceeds successfully without invoking the possible offensive from the host immune system (den Boon and Ahlquist, 2010). Upon infection, positive-strand RNA viruses create membranous replication organelles (ROs) by reorganizing host cell organelles. For instance, single-membrane invaginated vesicles of flavivirus and double-membrane vesicles (DMVs) of hepatitis C virus (HCV) and coronavirus stem from the endoplasmic reticulum (ER) (Knoops et al., 2008; Romero-Brey et al., 2012), whereas the DMVs or multilamellar vesicles of enterovirus originate from the ER and Golgi (Schlegel et al., 1996; Rust et al., 2001; Hsu et al., 2010; Limpens et al., 2011). The formation of ROs relies on virus proteins as well as host factors (Aktepe et al., 2017; Paul et al., 2018).

Flavivirus is a kind of positive-strand RNA virus, including yellow fever virus (YFV), dengue virus (DENV), Zika virus (ZIKV), West Nile virus (WNV), and so on. Millions of people are affected by flavivirus infection each year, posing a substantial threat to human health (Pierson and Diamond, 2020). Flaviviruses establish viral ROs on the host ER (Paul and Bartenschlager, 2015). The characteristic structures of flavivirus ROs are invaginated single-membrane vesicles and convoluted membranes. The invaginated vesicle is not completely sealed but connects with the cytosol through a narrow channel to exchange materials and products. Usually, several vesicles wrapped by the ER membrane constitute a vesicle packet (VP) (Welsch et al., 2009; Paul and Bartenschlager, 2015). Flavivirus replication is carried out by viral non-structural (NS) proteins, which are NS1, NS2A, NS2B, NS3, NS4A, NS4B, and NS5 (Apte-Sengupta et al., 2014). ER lumen located NS1 is a membrane-associated protein possessing multiple functions (Gutsche et al., 2011; Akey et al., 2014; Watterson et al., 2016). It has been shown that replication-required NS1 is located at the membranous replication site of flavivirus (Mackenzie et al., 1996; Lindenbach and Rice, 1997; Muylaert et al., 1997). Our previous work revealed that ZIKV NS1 is essential for the formation of ROs (Ci et al., 2020). NS1 remodels the host ER through its hydrophobic insertion into the ER membrane, creating invaginated vesicles that provide the environment for viral replication.

In the present work, we found that ZIKV NS1 preferentially binds to the negatively charged lipids, including PI4P. The positively charged residue R31 of NS1 is important for the NS1 association with negatively charged lipids on the ER membrane and NS1-induced ER remodeling in the cell. Downregulating phosphatidylinositol phosphates (PIPs) level by the overexpression of Sac1 or treatment with PI3K and PI4K inhibitors decreases NS1-induced ER remodeling and attenuates ZIKV replication. Our findings suggested that negatively charged lipids contribute to NS1-induced RO formation. This study provides a novel promising antiviral strategy targeting the electrostatic interaction between NS1 and negatively charged lipids.

## Results

### Non-structural Protein 1 Prefers to Bind to Negatively Charged Lipids

**FIGURE 1:**
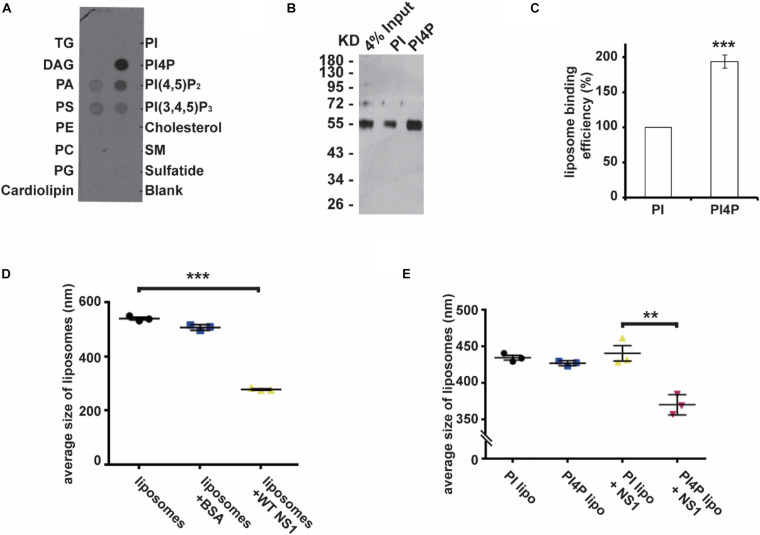
NS1 specifically binds to negatively charged lipids. **(A)** Lipid binding selectivity of ZIKV NS1. ZIKV Myc-NS1 proteins were incubated with a lipid membrane strip spotted with a series of different lipids. Antibody against the Myc tag was used to detect NS1 bound to each lipid spot. **(B)** NS1 preferentially binds to PI4P containing liposomes. The co-floating assay was performed to examine NS1 association with the liposomes. NS1 co-floated with the liposomes were collected from the top layer and analyzed by SDS-PAGE and silver staining. **(C)** Statistic analysis of NS1 co-floating efficiency in panel **(B)**. The amount of co-floating NS1 was analyzed. The amount of NS1 bound to PI-liposomes was set as 100%. **(D)** NS1 remodels the liposomes *in vitro*. After incubation with NS1, the average diameter of liposomes was detected by DLS. **(E)** PI4P containing liposomes are more sensitive to NS1-induced liposome remodeling. A less amount of NS1 was incubated with liposomes containing PI or PI4P. In the presence of PI4P, the average diameter of liposomes was decreased after incubation with NS1. The data are presented as the mean ± SEM. The *p*-values were obtained from a two-tailed *t*-test. **P < 0.01, ***P < 0.001.

Previous studies have revealed that flavivirus NS1 binds to and reshapes the lipid membrane by hydrophobic insertion (Akey et al., 2014; Ci et al., 2020). Our recent work had shown that ZIKV NS1 is the crucial factor to induce viral replication compartment. ER lumen localized NS1 remodeled ER structure to resemble the flavivirus ROs (Ci et al., 2020). To determine whether NS1 has different affinities to different lipids, we explored the binding selectivity of NS1 on a lipid strip and found that NS1 had a high affinity to negatively charged lipids including PI4P, PI(4,5)P_2_, PI(3,4,5)P_3_, PA, and PS (Figure 1A). This result suggests the possible role of specific interactions between NS1 and negatively charged lipids in the membrane binding and remodeling of NS1.

Given that PI4P has the highest affinity to NS1 among the lipids we screened, we introduced PI4P into liposomes and determined the influence of PI4P on the liposome-binding activity of NS1 by using a protein-liposome co-floating assay (Tucker et al., 2004). The co-floating assay is used to examine the protein-lipid membrane interaction. Membrane-associated proteins can be co-floated with liposomes via a density gradient ultracentrifugation, whereas unbound proteins stay at the bottom due to high buoyancy density. Purified NS1 proteins were incubated with liposomes with (w/) or without (w/o) PI4P, and then the NS1-liposome mixture was submitted to Nycodenz gradient centrifugation. The co-floating assay showed that NS1 binds to liposomes containing negatively charged PI4P more strongly than it does to those with phosphatidylinositol (PI, neutral) (Figures 1B,C). Akey et al. (2014) showed that NS1 remodeled liposomes *in vitro*. We measured the average diameter of the liposomes w/or w/o NS1 by DLS (dynamic light scattering) and found that NS1 led to a dramatic decrease in liposome diameter (Figure 1D). When we reduced the amount of NS1 incubated with liposomes, only PI4P-containing liposomes could be deformed by NS1 (Figure 1E), suggesting that negatively charged lipids increased the affinity of NS1 to the membrane and promoted the NS1 remodeling effect, which is meaningful for viral replication at the early stage when the expression of NS1 is limited.

### Positively Charged Contributes to the Electrostatic Interaction of NS1 With the Membrane

**FIGURE 2:**
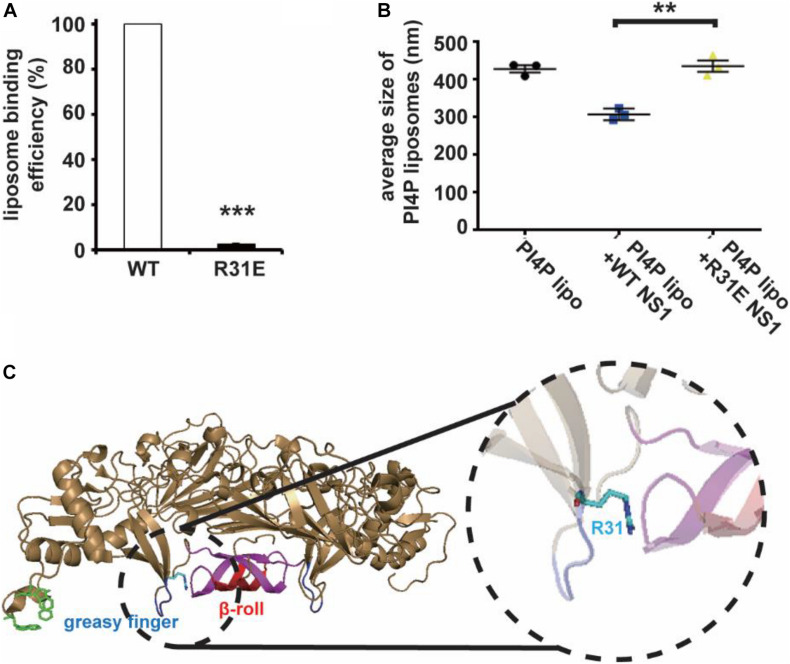
R31 of NS1 is important for the electrostatic interaction between NS1 and negatively charged lipids. **(A)** NS1 R31E mutant can hardly bind to liposomes containing PI4P. Positively charged arginine (R31) of NS1 was mutated to negatively charged glutamic acid (E). A co-floating assay was performed to examine the association of ZIKV NS1 with the liposomes. **(B)** NS1 R31E mutant lost the ability to remodel the PI4P liposomes. Liposomes containing PI4P were incubated with wild-type NS1 or R31E mutant. The average diameter of liposomes was detected by DLS. R31E mutant can hardly change the diameter of liposomes. **(C)** R31 located in the groove between β-roll and greasy finger on membrane association face. The data are presented as the mean ± SEM. The *p*-values were obtained from a two-tailed *t*-test. **P < 0.01, ***P < 0.001.

Non-structural Protein 1 has a higher affinity to negatively charged lipids, implying that the possibility of electrostatic interaction might be an important underlying mechanism of membrane binding and reshaping by NS1. We assessed the structure of NS1, searching for positively charged residues around the hydrophobic insertion region on the membrane binding face of NS1. Among several residues, we mutated some of them to negatively charged residues and found that the R31E mutation significantly decreased the ability of NS1 to bind to PI4P-containing liposomes (Figure 2A). Furthermore, the R31E mutation also impaired NS1-induced PI4P-liposome remodeling (Figure 2B). According to the crystal structure of NS1, R31 is located in the groove between two key hydrophobic regions of NS1, the β-roll and greasy finger (Figure 2C), which insert into the lipid membrane to induce membrane curvature (Akey et al., 2014; Brown et al., 2016; Xu et al., 2016). Thus, negatively charged lipids in the membrane are likely to interact electrostatically with the positively charged R31 residue in NS1. Therefore, this electrostatic interaction may stabilize and promote the association of NS1 with the membrane.

### Electrostatic Interaction Plays an Important Role in NS1-Induced ER Remodeling

**FIGURE 3:**
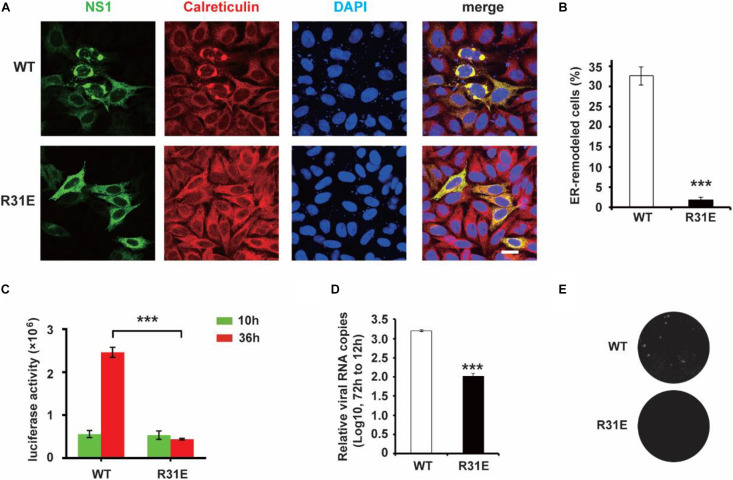
R31E mutation significantly impairs NS1 induced ER remodeling and attenuates viral replication. **(A)** ER morphology with the expression of WT and R31E mutant NS1. Wild type NS1 or R31E mutant with C-terminal Myc-tag was expressed in HeLa cells and then labeled with anti-Myc and anti-Calreticulin antibodies. WT NS1 expression induced apparent ER remodeling, whereas R31E mutant could rarely remodel the ER. Scale bar, 20 μm. **(B)** The percentage of ER-remodeled cells induced by expression of WT NS1 or R31E mutant. The ratio of ER-remodeled cells to total NS1 positive cells is defined as the percentage of ER-remodeled cells. **(C)** NS1 R31E mutation impairs viral replication. NS1 R31E mutation was introduced into the ZIKV replicon. Luciferase activity was measured at 10 h and 36 h post-transfection to validate the viral replication ability. **(D)** NS1 R31E mutation significantly impairs the production of infectious viruses. NS1 R31E mutation was introduced into ZIKV infectious clone. At about 72 h post-transfection, the culture medium was collected, and viral RNA was detected by RT-qPCR. **(E)** Plaque assay was performed using the recombinant ZIKV to infect Vero cells. The data are presented as the mean ± SEM. The *p*-values were obtained from a two-tailed *t*-test. ***P < 0.001.

Our previous work showed that ER lumen located NS1 induces ER remodeling to generate structures resembling flavivirus ROs (Ci et al., 2020). Next, we explored the role of electrostatic interaction between NS1 and negatively charged lipids in ER remodeling. Consistent with our previous results, wild-type NS1 induced apparent ER aggregation. However, the NS1 R31E mutant could hardly induce ER remodeling (Figures 3A,B), suggesting that electrostatic interaction between NS1 and negatively charged lipids is truly important for NS1-induced ER remodeling. To further investigate the effect of electrostatic interaction on viral replication, we mutated R31 of NS1 to glutamic acid (E) in the ZIKV replicon, which contains all replication-required NS proteins but lacks structural proteins. With *Renilla* luciferase as a reporter gene in the replicon, we evaluated the viral replication efficiency by detecting luciferase activity (Li et al., 2017). The similar luciferase activities of WT and R31E replicon at 10 h post-transfection indicated similar transfection and expression levels in the early stage. Compared to the wild-type replicon, R31E led to a significant reduction in viral replication at 36 h post-transfection due to the defect in viral replication (Figure 3C). Similarly, we introduced the R31E mutation into an infectious ZIKV clone and found that the R31E mutation significantly attenuated the production of infectious viruses (Figures 3D,E).

### PI4P Contributes to NS1-Induced RO Biogenesis

**FIGURE 4:**
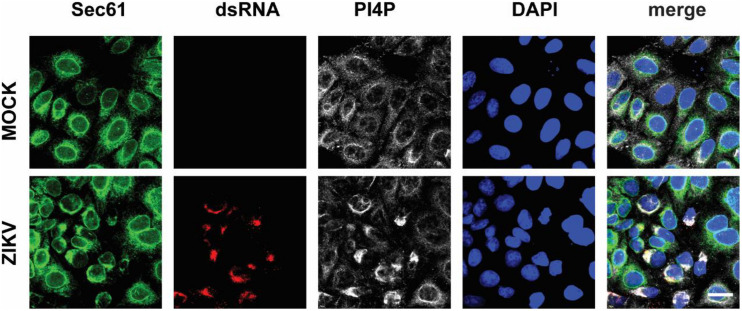
PI4P is enriched at ZIKV replication organelles. ZIKV infected EGFP-Sec61 stable cells were stained with antibodies against PI4P and dsRNA. Scale bar, 20 μm.

**FIGURE 5:**
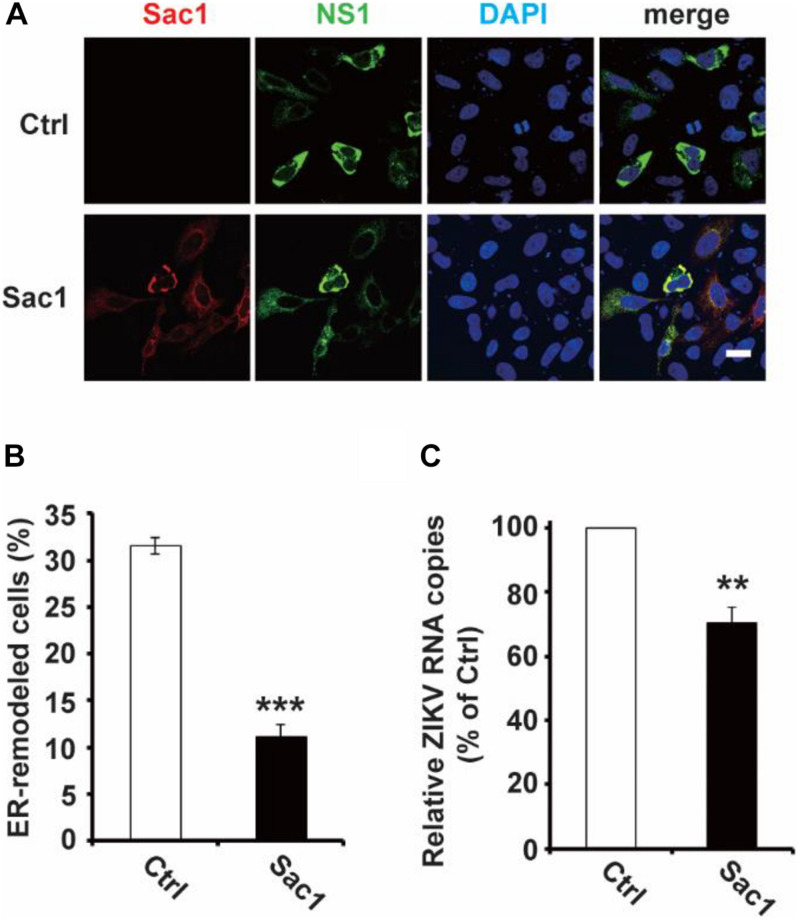
Sac1 overexpression interfered with NS1 induced ER remodeling and viral replication. **(A,B)** Sac1 overexpression interferes with NS1 induced ER remodeling. HeLa cells were co-transfected with Myc-NS1 and Flag-Sac1. Then cells were stained with anti-Myc and anti-Flag antibodies **(A)**. Scale bar, 20 μm. The ratio of ER-remodeled cells to Myc and Flag double-positive cells is calculated as the percentage of ER-remodeled cells **(B)**. **(C)** Sac1 overexpression impairs viral replication. BHK-21 cells were transfected with Sac1 expressing plasmid or control plasmid and then infected with ZIKV. The amount of viral RNA in the cell lysate at 48 hpi was quantified by RT-qPCR. The data are presented as the mean ± SEM. The *p*-values were obtained from a two-tailed *t*-test. ** *P* < 0.01, ***P < 0.001.

Next, we wondered whether PI4P localizes at the ZIKV replication site. To observe the location of PI4P, we marked PI4P by specific antibody in ZIKV infected Sec61 stable cells. We found that PI4P was enriched at the ROs, as indicated by viral dsRNA (Figure 4), suggesting that PI4P may be involved in viral replication. To explore the function of PI4P in ZIKV replication, we overexpressed the ER-associated PIP phosphatase Sac1 to downregulate PI4P and probably the levels of some other PIPs in the ER (Hsu et al., 2010; Zewe et al., 2018). We co-transfected NS1 and Sac1 into HeLa cells and observed the ER morphology. We found that Sac1 significantly decreased NS1-induced ER remodeling (Figures 5A,B). Furthermore, ZIKV replication was attenuated in cells overexpressing Sac1 (Figure 5C). Thus, the downregulation of PI4P, and other PIPs, can impair ZIKV replication by interfering with NS1-induced ER remodeling.

### PI Kinase Inhibitors Interfere With ZIKV Replication

**FIGURE 6:**
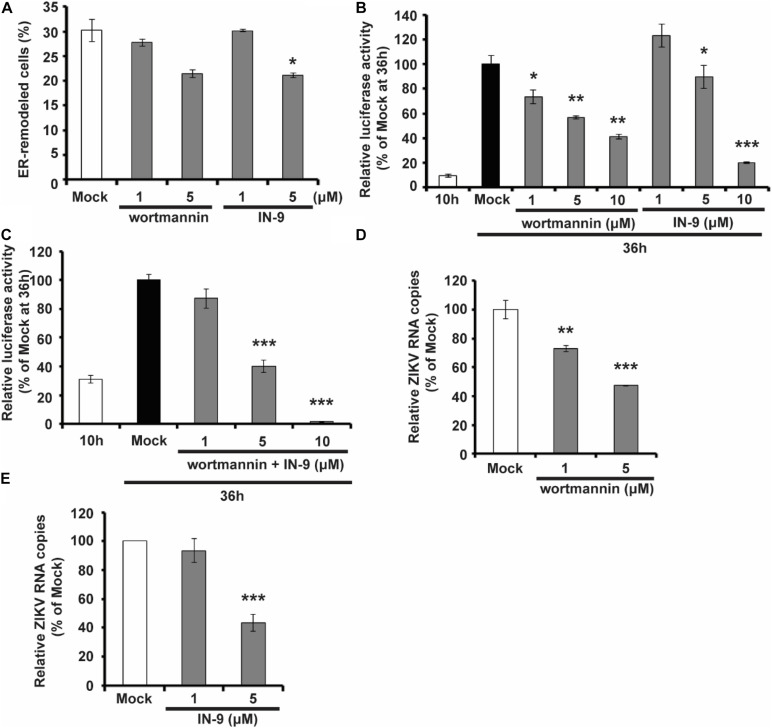
PIK inhibitors interfere with NS1 induced ER remodeling and viral replication. **(A)** PIK inhibitors decrease NS1 induced ER remodeling. NS1 expressed HeLa cells were treated with PIK inhibitors, wortmannin, or IN-9 at the indicated concentration. **(B)** Wortmannin or IN-9 inhibits ZIKV replication. BHK-21 cells were transfected by ZIKV replicon RNA. At 9 h post-transfection, cells were treated with indicated inhibitors. At 36 h post-transfection, luciferase activity was measured to evaluate viral replication. **(C)** The combination of Wortmannin and IN-9 shows improved inhibitory effects. ZIKV replicon RNA transfected BHK-21 cells were treated with Wortmannin and IN-9 at 9 h post-transfection. Luciferase activity was monitored at 36 h post-transfection. **(D,E)** Antiviral effects of PIK inhibitors. ZIKV infected BHK-21 cells were treated with Wortmannin **(D)** or IN-9 **(E)**. Viral RNA in cell lysate was quantified by RT-qPCR at 48 hpi. The data are presented as the mean ± SEM. The p-values were obtained from a two-tailed *t*-test. *P < 0.05, **P < 0.01, ***P < 0.001.

As shown above, NS1 prefers to bind to negatively charged lipids, including multiple PIP species, and PIPs can, in turn, affect the ability of NS1 to bind and remodel the membrane. PIPs are products of PI phosphorylation catalyzed by PI kinases. For example, PI3K and PI4K can phosphorylate PI to generate PI3P and PI4P, respectively. We then examined whether PI kinase inhibitors can interfere with ZIKV replication by altering PIP levels in the cell. We found that treatment with PI3K inhibitor Wortmannin and PI4K inhibitor PI4KIIIbeta-IN-9 decreased NS1-induced ER remodeling (Figure 6A). Moreover, based on a ZIKV replicon luciferase assay, both Wortmannin and IN-9 inhibited viral replication in a dose-dependent manner (Figure 6B). The combination of Wortmannin and IN-9 had an enhanced inhibitory effect. In the presence of 10 μM wortmannin and IN-9, we could hardly detect luciferase activity (Figure 6C). Finally, we measured the antiviral effects of these two inhibitors. Consistent with data from the ZIKV replicon assay, Wortmannin and In-9 attenuated ZIKV replication in a dose-dependent manner (Figures 6D,E). These findings suggest that downregulation of PIPs levels by inhibiting PI kinase activity may be a new strategy to interfere with NS-induced ER remodeling and inhibit viral replication.

### Electrostatic Interaction Between Negatively Charged Lipids and NS1 Is Common to Various Flaviviruses

**FIGURE 7:**
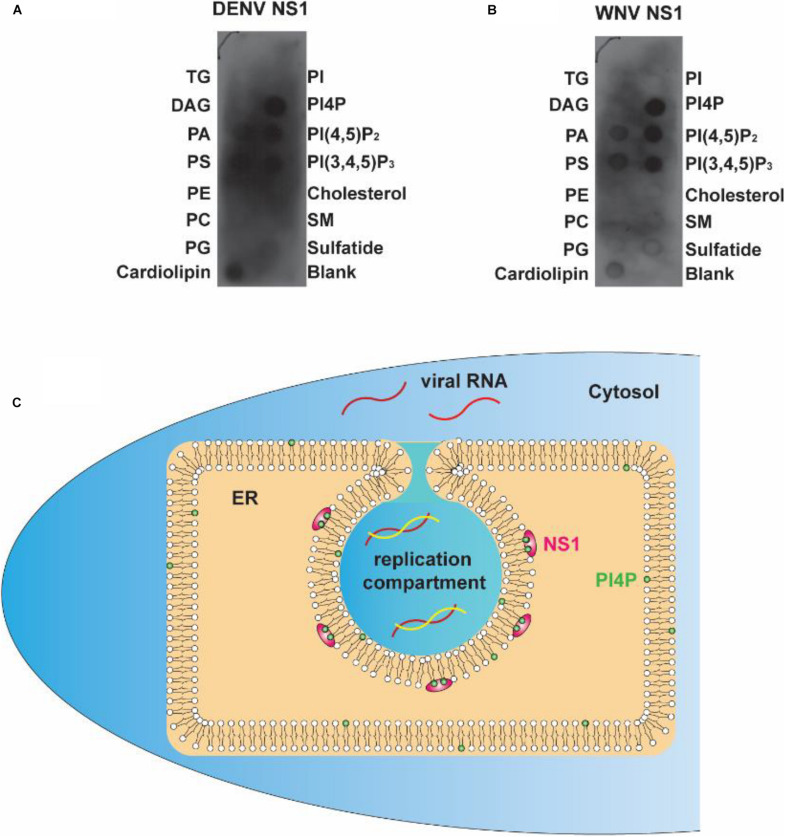
Flavivirus shares a common RC generation mechanism. **(A,B)** DENV and WNV NS1 possessed a similar binding pattern to negatively charged lipids as ZIKV NS1. Lipid membrane strips were used to examine the lipid-binding selectivity of DENV **(A)** and WNV NS1 **(B)**. **(C)** Flaviviral NS1 proteins located in the ER lumen bind to the inner leaflet of the ER membrane by hydrophobic insertion, and the electrostatic interaction with negatively charged lipids (e.g., PI4P) facilitates the association between NS1 and membrane. This insertion of NS1 changes the ER membrane’s curvature, producing invaginated vesicles as the place of viral RNA synthesis, the replication compartment.

To determine whether the electrostatic interaction between NS1 and negatively charged lipids is a common rule shared by flaviviruses, we explored the affinity of DENV and WNV NS1 to different lipids. Using a lipid strip blot assay, we found that DENV and WNV NS1 showed similar binding patterns to ZIKV NS1. PI4P was the strongest binding target, followed by PI(4,5)P_2_, PI(3,4,5)P_3_, and then PS and PA (Figures 7A,B). This finding suggested that electrostatic interaction between negatively charged lipids and NS1 may be a common mechanism for NS1-induced RO formation among flaviviruses.

The present work and our earlier study argue for a crucial role of NS1 in flavivirus RO biogenesis. Viral NS1 remodels the ER membrane by hydrophobic insertion, and its electrostatic interaction with negatively charged lipids facilitates the process (Figure 7C). Other NS proteins may also participate in the construction of ROs for viral RNA replication.

## Discussion

As the place of replication complex assembly and viral RNA synthesis, ROs are prerequisite structures for flavivirus replication. The underlying mechanism of RO formation is under discussion (Roosendaal et al., 2006; Miller et al., 2007; Plaszczyca et al., 2019; Cerikan et al., 2020). Our recent work has elucidated the crucial role of NS1 in RO formation. Here we revealed that the electrostatic interaction between NS1 and negatively charged lipids is important for NS1-induced ER remodeling. We found that NS1 has the highest affinity to PI4P and that PI4P is enriched at ZIKV ROs, indicating the involvement of PI4P in flavivirus replication. Other negatively charged lipids, such as PI(4,5)P_2_ and PI(3,4,5)P_3_, have lower affinity to NS1 than PI4P, probably because of the steric hindrance from the larger head group of PI(4,5)P_2_ or PI(3,4,5)P_3_, which can barely fit into the groove between the β-roll and greasy finger of NS1. On the other hand, PI(4,5)P_2_ and PI(3,4,5)P_3_ are mostly located on the plasma membrane, not the ER membrane (Heo et al., 2006). Therefore, PI(4,5)P_2_ and PI(3,4,5)P_3_ may contribute less to flavivirus RO biogenesis.

PIPs are important lipids involved in many signaling pathways and membrane trafficking. Most of these processes are mediated by the specific recognition of PIPs by proteins containing PIP-binding domains, such as the FYVE domain, pleckstrin homology (PH) domain, and phox (PX) domain (Harlan et al., 1994; Garcia et al., 1995; Ponting, 1996; Seaman et al., 1998; Misra and Hurley, 1999; Dumas et al., 2001; Kanai et al., 2001; Song et al., 2001; Ellson et al., 2002; Kutateladze, 2006). On the other hand, electrostatic interactions between the negatively charged head groups of PIPs and proteins play roles in this protein-lipid interaction (Lai et al., 2012). NS1 has less strict binding specificity to negatively charged lipids, which is different from those mentioned above. The positively charged residues on the membrane-association face of NS1, for example, R31, play an important role in NS1-PIPs interaction. However, the structural basis of the selective binding of NS1 to different lipids remains to be explored.

As positive-strand RNA viruses replicate in membranous compartments, PIPs are widely involved in virus replication. Vps34 recruitment-medicated PI3P enrichment is involved in RO formation of tomato bushy stunt virus (TBSV) and carnation Italian ringspot virus (CIRV) on the peroxisomal membrane and mitochondrial membrane, respectively (Feng et al., 2019). Enteroviral 3A protein recruits PI4KIIIβ to catalyze PI4P production from PI, leading to PI4P enrichment at ROs to regulate viral RNA replication (Hsu et al., 2010). Similarly, HCV infection-induced PI4P increase and localization at ROs are important regulators of viral replication (Hsu et al., 2010). Our present work reveals that PIPs also affect flavivirus replication by modulating ROs formation. This finding also provides a new strategy for interfering with flavivirus replication. The downregulation of PI4P (and probably other PIPs) on the ER membrane by overexpressing Sac1 or treating the cell with PI3K and PI4K inhibitors decreased NS1-induced ER remodeling and attenuated ZIKV replication.

In summary, our findings highlight the importance of the electrostatic interaction between NS1 and negatively charged lipids in flavivirus RO formation, which enriches our understanding of the mechanism of flavivirus replication. The involvement of PIPs in flavivirus RO also allows us to develop a novel antiviral strategy by regulating PIPs metabolism.

## Materials and Methods

### Cell Culture

Human HEK293T cells (embryonic kidney cells, ATCC CRL3216), human HeLa cells (ATCC CCL-2), and Vero cells (African Green Monkey kidney cells, ATCC CCL-81) were grown at 37°C with 5% CO_2_ in DMEM with 10% FBS. BHK-21 cells (baby hamster kidney fibroblast cells, ATCC CCL10) were grown at 37°C with 5% CO_2_ in DMEM with 5% FBS.

### Antibodies

Antibodies used in the present study include anti-Myc (MBL, M192-3), dsRNA-J2 (Scicons), anti-Flag (Cell Signaling Technology, 14793), Anti-PI4P (Z-P004, Echelon Biosciences) Alexa 488-conjugated goat anti-mouse antibody (Invitrogen), and Cy3-conjugated Goat anti-Rabbit antibody (Jackson Immuno Research).

### Non-structural Protein 1 Protein Purification

Non-structural protein 1 with N-terminal Myc and C-terminal His-tag coding sequence was constructed into pcDNA 3.1 after bovine pre-prolactin signal sequence (ss) (MDSKGSSQKGSRLLLLLVVSNLLLCQGVVST), which can lead to NS1 expression in the ER lumen. NS1 proteins were purified as previously described (Plaszczyca et al., 2019). Briefly, 293T cells were transfected with ss-NS1 expressing plasmid. NS1 can be secreted into the culture media. At 24 h post-transfection, refresh the media with Opti-MEM medium, and the cells were cultured for another 48 h. Collect the media and centrifuge to remove suspending cells. Nickel-magnetic beads (Selleck) were used to purify His-tagged NS1 proteins from the medium.

### Lipid Strip Binding Assay

The lipid-binding specificity of NS1 was identified by membrane lipid strip (Echelon Biosciences Inc., p-6002). In brief, the strip was blocked by 3% BSA in TBST. Then the membrane strip was incubated with 0.5 ug Myc-tagged NS1 proteins for 1 h. After three washes, the anti-Myc antibody (1:5,000) was incubated with the strip for 1 h, followed by the secondary antibody incubation for another 1 h. Lipid-bound NS1 proteins were detected by ECL plus western blot system (Perkin Elmer, Waltham, MA, United States).

### Zika Virus Infection

BHK-21 cells were infected by ZIKV (MOI 0.1). After 2 h, remove media containing viruses and refresh with complete media. Cells were cultured for indicated times.

### Liposome Preparation

Liposomes were prepared as previously described. DOPC, Cholesterol, and PI/PI4P (dissolved in chloroform) were mixed at a molar ratio of 85:10:5. The lipid mixture was dried by nitrogen following vacuumed for 2 h to remove chloroform completely. Resuspend lipid with 800 ul buffer A (100 mM NaCl, 50 mM Tris at pH 7.5) and vertex for 30 min. After seven cycles of freeze-and-thaw, extrude the liposomes through 100 or 800 nm polycarbonate membrane filter (Whatman, 610005 and 610009) with Avanti Mini-Extruder (Avanti) 21 times.

### Liposome Co-floating Assay

Non-structural protein 1 proteins were incubated with 100 nm liposomes (NS1: liposome, 1,000:1) at 37°C for 4 h. the liposome-protein mixture was then submitted to density gradient centrifugation. 150 μl liposome-protein mixture was mix with an equal volume of 80% Nycodenz. Then the mixture was transferred to the centrifuge tube, followed by 250 μl buffer A (100 mM NaCl and 50 mM Tris at pH 7.5) containing 30% Nycodenz and then 50 μl buffer without Nycodenz on the top. Samples were centrifuged at 4°C, 40,000 rpm for 4 h. 75 μl sample was collected from the top as a co-floating sample after the ultracentrifugation.

### Liposome Remodeling Assay

A total of 10 μl liposomes (extruded through 800 nm filter) were mixed with 40 μl buffer A (100 mM NaCl and 50 mM Tris at pH 7.5) or 40 μl 3 mg/ml NS1 or BSA (negative control) proteins and then incubated at 37°C for 2 h. In the PI4P-liposome remodeling assay, 15 μl 3 mg/ml NS1 was used. The average size of liposomes was determined by dynamic light scattering. The liposome-protein mixture was transferred to ZEN0040 cuvette, and the average size of liposomes was measured by Zetasizer Nano ZS90.

### PI4P Labeling in ZIKV Infected Cells

HeLa cells stably expressing Sec61β were infected with ZIKV. At 48 hpi, cells were fixed with 2% PFA and then permeabilized and blocked with 0.1% digitonin diluted in PBS containing 1% BSA for 1 h. Then, Cells were stained with antibodies against PI4P and dsRNA at 4°C overnight.

### Zika Virus Replicon

Zika virus replicon was gifted from Dr. Zhang Bo at the Institute of Virology, Wuhan, Chinese Academy of Sciences. The R31E mutation was constructed by QuikChange Site-Directed Mutagenesis Kit. ZIKV replicon luciferase assay was performed as previously described (Plaszczyca et al., 2019). ZIKV replicon RNA was transcribed *in vitro* using mMESSAGE mMACHINE T7 transcription kit (Ambion, Thermo Fisher Scientific). BHK-21 cells were transfected with replicon RNA by Viafect (Promega), and cells were lyzed at 10 or 36 h post-transfection. Luciferase assay was performed by the *Renilla* luciferase assay system (Promega).

### Recombinant ZIKV Production

Zika virus infectious clone was kindly gifted from Dr. Peiyong Shi, University of Texas Medical Branch, Galveston, TX, United States. The R31E mutation was constructed by QuikChange Site-Directed Mutagenesis Kit, and the full-length ZIKV RNA was transcribed *in vitro* using mMESSAGE mMACHINE T7 transcription kit (Ambion, Thermo Fisher Scientific). The yield RNAs were transfected into BHK-21 cells by Viafect (Promega). The culture media containing recombinant ZIKV was harvested at day 3 post-transfection.

### Plaque Assay

Vero cells were seeded into a 12-well plate. The medium containing recombinant ZIKV was 1:10 dilute in DMEM containing 2% FBS and then added to Vero cells. After 2 h incubation, remove the media and add 1 ml 1% low-melting agarose. After another 4 days, cells were fixed with 4% formaldehyde for 1 h and stained by 1% crystal violet.

### Statistical Analysis

A minimum of three biological repeats was performed for each experiment. The statistical significance between the two groups’ data (normal distribution) was determined by a two-tailed *t*-test. The normality testing uses the Shapiro-Wilk test.

## Data Availability Statement

The original contributions presented in the study are included in the article/supplementary material, further inquiries can be directed to the corresponding authors.

## Author Contributions

YC and YY performed the experiments. YC, CX, C-FQ, and LS designed the project and analyzed the data. YC and LS wrote the manuscript together with all other authors. All authors contributed to the article and approved the submitted version.

## Conflict of Interest

The authors declare that the research was conducted in the absence of any commercial or financial relationships that could be construed as a potential conflict of interest.
